# Seasonal induction of alternative principal pathway for rose flower scent

**DOI:** 10.1038/srep20234

**Published:** 2016-02-01

**Authors:** Hiroshi Hirata, Toshiyuki Ohnishi, Kensuke Tomida, Haruka Ishida, Momoyo Kanda, Miwa Sakai, Jin Yoshimura, Hideyuki Suzuki, Takamasa Ishikawa, Hideo Dohra, Naoharu Watanabe

**Affiliations:** 1 Graduate School of Science and Technology, Shizuoka University, 836 Ohya, Suruga-ku, Shizuoka 422-8529, Japan; 2 Graduate School of Agriculture, Shizuoka University, 836 Ohya, Suruga-ku, Shizuoka 422-8529, Japan; 3 Research Institute of Green Science and Technology, Shizuoka University, 836 Ohya, Suruga-ku, Shizuoka 422-8529, Japan; 4 United Graduate School of Agricultural Science, Gifu University (Shizuoka University), 836 Ohya, Suruga-ku, Shizuoka 422-8529, Japan; 5 Graduate School of Science and Technology, Shizuoka University, 3-5-1 Johoku, Naka-ku, Hamamatsu 432-8561, Japan; 6 Department of Environmental and Forest Biology, State University of New York College of Environmental Science and Forestry, Syracuse, New York, 13210, USA; 7 Marine Biosystems Research Center, Chiba University, Kamogawa, Chiba, 299-5502, Japan; 8 Graduate School of Engineering, Shizuoka University, 3-5-1 Johoku, Naka-ku, Hamamatsu 432-8561, Japan; 9 Kazusa DNA Research Institute, Kisarazu, Chiba 292-0818, Japan; 10 Human Metabolome Technologies Inc., Tsuruoka, Yamagata 997-0052, Japan

## Abstract

Ecological adaptations to seasonal changes are often observed in the phenotypic traits of plants and animals, and these adaptations are usually expressed through the production of different biochemical end products. In this study, ecological adaptations are observed in a biochemical pathway without alteration of the end products. We present an alternative principal pathway to the characteristic floral scent compound 2-phenylethanol (2PE) in roses. The new pathway is seasonally induced in summer as a heat adaptation that uses rose phenylpyruvate decarboxylase (RyPPDC) as a novel enzyme. *RyPPDC* transcript levels and the resulting production of 2PE are increased time-dependently under high temperatures. The novel summer pathway produces levels of 2PE that are several orders of magnitude higher than those produced by the previously known pathway. Our results indicate that the alternative principal pathway identified here is a seasonal adaptation for managing the weakened volatility of summer roses.

Seasonal adaptations are observed for various animal and plant traits, including changes to the flowering period in many plants and adaptations to cold climates in fish, mammals, and birds^1^,^2^,^3^,^4^,^5^,^6^. Certain plants begin developing flower buds under specific combinations of ambient temperature and photoperiods, whereas mammals (birds) may experience changes to their fur (feathers) in the fall in preparation for the colder temperatures of winter. In these morphological and physiological adaptations, biosynthetic pathways are shifted to another route, leading to a different end product.

As compared with seasonal adaptations that lead to different end products, some plants and animals decrease their level of chemical reactions under environmental stresses, resulting in a reduction of end products. For instance, photosynthesis is reduced by high light stress under the stronger light and higher temperature conditions of the summer^7^,^8^,^9^. The expression of genes such as that encoding isoprene synthase also varies with seasonal changes in ambient temperature^10^. These responses to environmental stress usually involve interference with a particular enzyme in a given biosynthetic pathway.

In certain biochemical processes, the essential metabolites are often associated with multiple synthetic pathways or bypasses that compensate for disturbances in the major pathway, and the interruption of certain steps in the pathways may be induced by genetic mutations or environmental stresses. To ensure the production of essential metabolites, the disturbed pathway is re-routed to a bypass or an alternative pathway^11^,^12^,^13^. In such cases, the disruption of metabolite production is usually lethal. As compared with essential metabolites, plant secondary products, such as a unique scent or flower color pigment, are often associated with a single biosynthetic pathway, and genetic alterations or mutations of one of the associated genes can easily inhibit the production of these products completely^14^,^15^. Therefore, an adaptive change in the biochemical pathway itself (without altering the end product) would be generally unexpected.

The aroma volatiles emitted from rose flowers to attract pollinators^16^,^17^ primarily consist of terpenes, aromatic compounds, and fatty acid derivatives^18^. One of the dominant scent compounds emitted by Damask roses such as *Rosa* × *damascena*, *R*. *x hybrida* ‘Hoh-Jun’, and *R*. *x hybrida* ‘Yves Piaget’ (Fig. 1a) is 2-phenylethanol (2PE), which is a common flavor and fragrance ingredient in cosmetics and perfumes^19^,^20^,^21^. 2PE is biosynthesized in roses from L-phenylalanine (L-Phe) *via* two sequential enzymatic reactions catalyzed by aromatic amino acid decarboxylase (AADC)^22^,^23^ and phenylacetaldehyde reductase (PAld reductase: PAR)^24^ (Fig. 1b).

In this study, we report on the seasonal summer induction of an alternative principal pathway for the production of 2PE from L-Phe. This pathway becomes active only in summer under high-temperature conditions (Fig. 1b), in contrast to the previously established pathway, which is active throughout the year, regardless of temperature, and produces a nearly constant amount of 2PE. However, the amount of 2PE produced in summer by the alternative principal pathway is increased several-fold over that of the established pathway. Thus, the second pathway is a seasonal adaptation for hot summer conditions, and it involves the first identified plant phenylpyruvic acid (PPA) decarboxylase (PPDC). Here, we discuss the alternative principal pathway in relation to adaptations to summer environmental conditions and discuss whether this type of alternative pathway is available for plant secondary metabolites.

## Results

### Differences in the [^2^H_8_]/[^2^H_7_]-2PE ratios produced from L-[^2^H_8_]Phe in rose flowers

**Figure 1:**
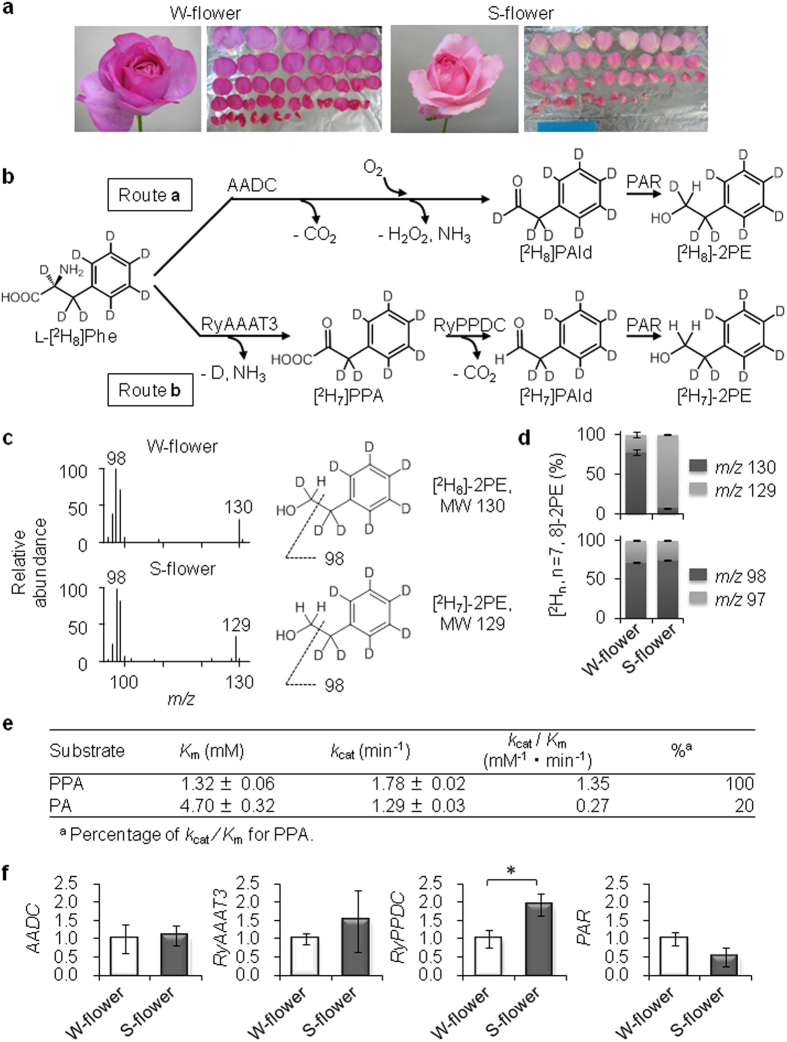
Two alternative principal pathways leading to synthesis of the major floral scent compound 2PE in roses and their activities in winter and summer flowers. [^2^H_8_]-2PE and [^2^H_7_]-2PE from L-[^2^H_8_]Phe in rose petals, and a comparison of activities in winter and summer flowers. (**a**) Photo of a representative W-flower and S-flower (left) and individual petals (right). (**b**) Two alternative principal pathways leading to 2PE. In W-flowers (route a), [^2^H_8_]PAld is synthesized by AADC after a Schiff base formation, the release of carbon dioxide, and the retention of the α-deuterium of L-[^2^H_8_]Phe. In S-flowers (route b), RyAAAT3 forms a Schiff base and releases α-deuterium prior to decarboxylation; [^2^H_7_]PPA is then converted to [^2^H_7_]PAld by PPDC. In both routes, [^2^H_n_, n = 7, 8]PAld is converted to 2PE by PAR. (**c**) Typical mass spectra of [^2^H_n_, n = 7, 8]-2PE produced by rose protoplasts of W-flowers harvested in January (upper) and S-flowers harvested in August (lower). (**d**) Ratios of [^2^H_n_, n = 7, 8]-2PE synthesized in the L-[^2^H_8_]Phe feeding experiments in protoplasts prepared in W-flower (left) and S-flower (right). The production ratios of [^2^H_8_]- and [^2^H_7_]-2PE were calculated according to the intensities of the molecular ions at *m/z* 130 and *m/z* 129 (upper diagram). The ratios for the benzyl cations (lower diagram) at *m/z* 98 and *m/z* 97 were used to determine the chemical structure of each isotopolog. Error bars represent the standard deviation (SD) (n = 3). (**e**) Kinetic parameters of RyPPDC. Mean ± SD (n = 5). PPA and PA indicate phenylpyruvic acid and pyruvic acid, respectively. (**f**) Comparison of transcript levels involved in the 2PE pathway in W-flowers and S-flowers. Transcript analysis of the biosynthetic enzyme genes for 2PE in W-flowers (white bars) and S-flowers (gray bars) was performed by real-time PCR. All of the genetic data were standardized to β-actin transcript levels and represent the relative transcript level. The transcript levels of W-flowers were set to 1.0. Error bars represent the standard error (SE) (n = 5); *indicates *P* < 0.05.

When L-[^2^H_8_]Phe is administered to intact rose flowers, [^2^H_8_]-2PE is detected as a dominant isotopologue, indicating that it is derived from L-[^2^H_8_]Phe with retention of the α-position deuterium atom^23^. In recent years, however, we have repeatedly detected [^2^H_7_]-2PE in intact rose flowers during one specific season. To investigate the two different pathways, we first compared the 2PE synthetic activity in rose protoplasts harvested in January (winter (W)-flower) and in August (summer (S)-flower) (Fig. 1c,d). The typical mass spectra indicated that [^2^H_8_]-2PE (*m/z* 130 [M]^+^) was the major compound in W-flowers, whereas [^2^H_7_]-2PE (*m/z* 129 [M]^+^) was the major component in S-flowers (Fig. 1c). A feeding experiment with L-[^2^H_8_]Phe also revealed that the dominant isotopolog was [^2^H_8_]-2PE in W-flowers and [^2^H_7_]-2PE in S-flowers (Fig. 1d). In both cases, the benzyl cation [^2^H_n_, n = 7, 8]-2PE (*m/z* 98 [C_7_^2^H_7_]^+^) was the dominant isotopolog. The ratio of [^2^H_8_]-2PE to [^2^H_7_]-2PE was calculated on the basis of the ion intensity ratio of *m/z* 130 [M^+^] of [^2^H_8_]-2PE to *m/z* 129 [M^+^] of [^2^H_7_]-2PE. The molecular ion ratios were calculated as follows: [^2^H_8_]/[^2^H_7_]-2PE = 78.2 ± 3.4/21.8 ± 3.4 (W-flower) and [^2^H_8_]/[^2^H_7_]-2PE = 7.9 ± 0.6/92.1 ± 0.6 (S-flower) (Fig. 1d). Thus, the protoplast study showed that [^2^H_7_]-2PE was dominant in S-flowers. In addition, a feeding experiment with L-[^2^H_8_]Phe and intact S-flowers of *R*. *x hybrida* ‘Yves Piaget’ in August produced an [^2^H_8_]/[^2^H_7_]-2PE ratio of 31.4 ± 3.2/68.6 ± 5.0, whereas the corresponding ratio in W-flowers in February was 67.8 ± 11.5/32.2 ± 10.9. Similar results were observed for the S-flowers of *R. x hybrida* ‘Hoh-Jun’. These results indicate that the dominant product [^2^H_7_]-2PE is produced by the loss of ^2^H at the α-position (C-2) of L-[^2^H_7_]Phe in S-flowers (Fig. 1b). Thus, the seasonal pathway for 2PE (route **b**) is common in rose flowers (Fig. 1).

Route **a** (W-flower) of the 2PE biosynthetic pathway has previously been confirmed (Fig. 1b)^22^,^23^. In route **a**, L-[^2^H_8_]Phe is converted by AADC after the formation of the Schiff base by the removal of the carboxyl group to [^2^H_8_]PAld and the retention of an α-position deuterium. [^2^H_8_]PAld is then catalyzed by PAR to [^2^H_8_]-2PE. The finding of [^2^H_7_]-2PE dominance in August suggests the presence of an alternative principal pathway for 2PE, which is route **b** in S-flowers. Here, the last step in route **b** is also the catalysis of [^2^H_7_]PAld to [^2^H_7_]-2PE by PAR. We recently found that rose aromatic amino acid aminotransferase 3 (RyAAAT3) converts L-[^2^H_8_]Phe to [^2^H_7_]PPA without the retention of an α-position deuterium, which is inconsistent with AADC in route **a**^25^. Therefore, the missing link in route **b** is the conversion of [^2^H_7_]PPA to [^2^H_7_]PAld by PPDC^25^,^26^,^27^.

### Enzyme catalyzing PPA decarboxylation identified as rose PPDC

The plant PPDC in rose (RyPPDC) was identified to catalyze the decarboxylation of PPA as described below (Fig. 1b). First, PPDC was purified from the S-flower petals (Supplementary Fig. 1a,b), and gas chromatography mass spectrometry (GC-MS) was used to trace PAld production from PPA, resulting in identification of partial amino acid sequences (Supplementary Table 1). Heterologously expressed RyPPDC from a baculovirus–insect cell system was used to catalyze PPA decarboxylation (Supplementary Fig. 1c,d), and the *k*_cat_/*K*_m_ value of RyPPDC was higher for PPA than for pyruvic acid (PA) (Fig. 1e). Thus, we confirmed that RyPPDC converts PPA to 2PE *via* PAld in the novel pathway (Fig. 1b).

To verify the structure of route **b**, we analyzed the transcript levels of biosynthetic enzymes in the 2PE pathway by real-time PCR (Fig. 1b). The *RyPPDC* transcript level was significantly higher in S-flowers than in W-flowers, but *AADC*, *RyAAAT3* and *PAR* transcript levels did not differ between S- and W-flowers (Fig. 1f). These results show that *RyPPDC* contributes to the conversion of PPA to [^2^H_7_]-2PE *via* route **b** in S-flowers.

### Seasonal change in the 2PE pathway in *R*. *x hybrida* ‘Yves Piaget’

**Figure 2:**
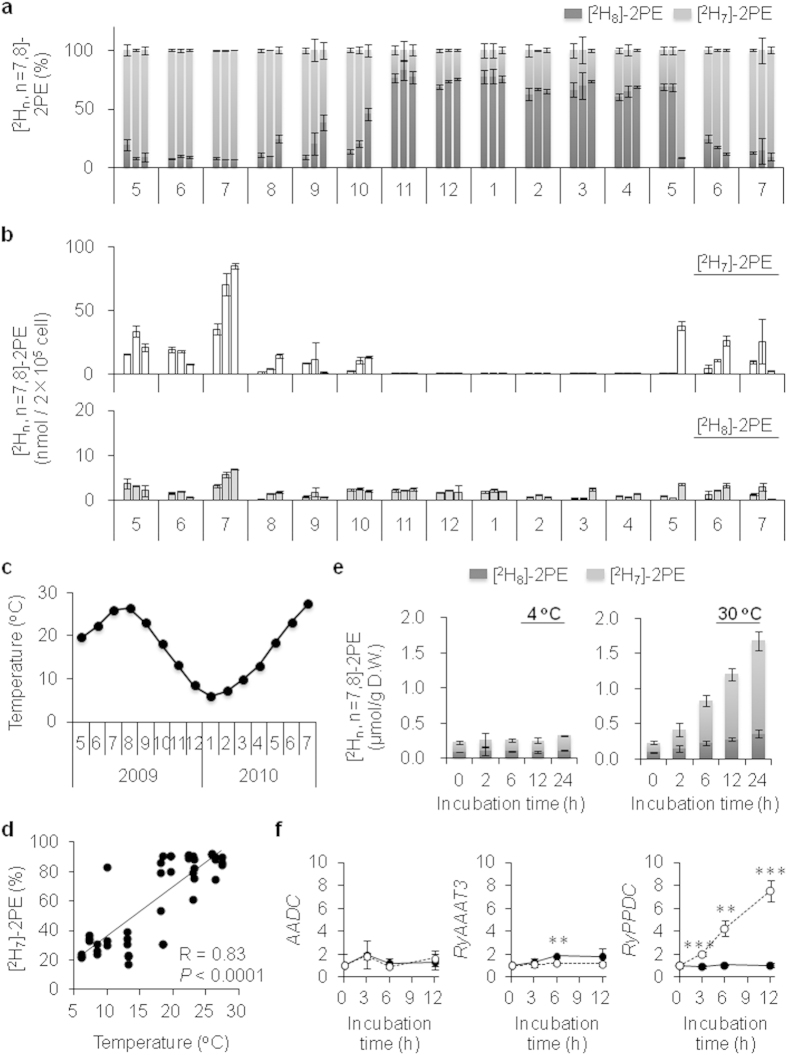
Seasonal changes in the production of [2Hn, n = 7, 8]-2PE and its. 11 biosynthetic enzymes. (**a**,**b**) Seasonal changes in the [2H8]- and [2H7]-2PE ratios (**a**) and amounts (**b**) in protoplasts collected each month for 15 months (month 1 is January). Each month, three flowers were used to prepare three independent protoplast samples. The protoplasts were fed with L-[2H8]Phe as described in the Methods. Error bars represent the SD (n = 3). The rose used was *R*. *x hybrida* ‘Yves Piaget’. Note that the y-axis scale is different for [2H8]- and [2H7]-2PE in **b**. (**c**) Seasonal changes in the average temperature in Mishima City. All of the data were obtained from the Mishim Local Meteorological Observatory. (**d**) Relationship between the [2H7]-2PE ratio and average temperature. (**e**) Production of [2Hn, n = 7, 8]-2PE at 4 °C, and at 30 °C. (**f**) Changes in 2PE-related gene transcript levels in temperature-treated rose petals. All of the genetic data were standardized to β-actin transcript levels and represent the relative transcript amount. Each transcript was quantified from three independent reverse transcription reactions. The transcript levels before incubation were set to 1.0. Closed circles and open circles depict low-temperature (4 °C) and high-temperature (30 °C) treatment, respectively. Error bars represent the SD (n = 3). **P < 0.01 and ***P < 0.001 indicate values that were significantly different.

Next, we characterized the seasonal variation in the synthesis of these 2PE isotopologs (Fig. 2) by administering L-[^2^H_8_]Phe to floral protoplasts extracted from three independent flowers of *R*. *x hybrida* ‘Yves Piaget’ harvested in each month from May 2009 to July 2010 (Fig. 2a,b). [^2^H_7_]-2PE was the dominant species detected among the [^2^H_n_, n = 7, 8]-2PE isotopologs from May/June to October, whereas [^2^H_8_]-2PE was dominant from November to April (Fig. 2a). [^2^H_8_]-2PE was synthesized at a constant level throughout the year; by contrast, [^2^H_7_]-2PE was synthesized at levels 5-to-12-fold higher than that of [^2^H_8_]-2PE from June to October, and was nearly absent from November to April (Fig. 2b). These results suggest that the route **b** pathway is seasonally activated in rose flowers in summer, whereas route **a** is maintained throughout the year.

### A key factor in the seasonal shift in 2PE biosynthesis

The seasonal changes in temperatures (Fig. 2c) correlated with the changes in [^2^H_7_]-2PE ratio (Fig. 2d). To evaluate the effects of temperature on [^2^H_n_, n = 7, 8]-2PE production, rose flowers were incubated at low (4 °C) and high (30 °C) temperatures. During incubation at 4 °C, the amounts of [^2^H_8_]- and [^2^H_7_]-2PE did not change, whereas at 30 °C, the production of [^2^H_7_]-2PE increased more markedly (9.5-fold) as compared with [^2^H_8_]-2PE (3.9-fold) (Fig. 2e). These results suggest that [^2^H_7_]-2PE biosynthesis in rose petals is activated by high-temperature incubation.

Transcript analyses of the 2PE biosynthetic genes also revealed that the relative transcript levels of *AADC* did not change and those of *RyAAAT3* remained constant, even at 30 °C; in addition, transcript levels were slightly lower under incubation at 30 °C than under incubation at 4 °C (Fig. 2f). However, *RyPPDC* transcripts were significantly increased in a time-dependent manner under incubation at 30 °C, indicating that [^2^H_7_]-2PE production at high temperatures is activated by increased transcript levels of *RyPPDC*. Thus, the significant increase in *RyPPDC* transcripts and the resulting [^2^H_7_]-2PE indicate that temperature is a key factor in the seasonal shift in 2PE biosynthesis.

## Discussion

The current results confirmed that an alternative principal pathway (route **b**) is highly active in summer (S-flowers). In this route, RyAAAT3 converts L-[^2^H_8_]Phe to [^2^H_7_]PPA^25^, which is then converted by RyPPDC to [^2^H_7_]PAld, and then to [^2^H_7_]-2PE by PAR (Fig. 1b). In a previous study, Sakai *et al.* reported that PAR immediately converts [^2^H_n_, n = 7,8]PAld to [^2^H_n,_ n = 7,8]-2PE without a change in deuterium number or position^22^. Hence, the [^2^H_n,_ n = 7,8]-2PE ratio in W- and S-flowers would reflect the [^2^H_n,_ n = 7,8]PAld ratio. This summer pathway is principally induced by high temperature, which leads to an up-regulation of RyPPDC (Fig. 2). Roses are generally grown in temperate climates, and the summer season in Japan is characterized by higher temperatures and longer periods of light. In addition to temperature, a significant correlation was observed between the [^2^H_7_]-2PE ratio and day time length recorded in Mishima city, Japan, where the rose flowers were grown (Fig. 2c,d, Supplementary Fig. 2a and b). High temperature is most probably a key factor in the seasonal pathway of 2PE, and day length is also likely to be involved in it. Short-term high temperatures affected 2PE biosynthesis in addition to the long-term conditions of the summer season. A concomitant increase in [^2^H_7_]-2PE production and *RyPPDC* transcript levels was observed with high-temperature incubation, whereas no changes were observed at 4 °C (Fig. 2e,f). These results suggest that the novel pathway is activated by short-term high temperature and that the heat induction of *RyPPDC* is likely to be a key step in this pathway. Furthermore, the biosynthetic enzymes in the pathway in rose flower petals seem to be inactive at 4 °C.

The question of why roses activate 2PE biosynthesis using a novel pathway in summer remains. We found that there was (1) a decrease in petal growth in S-flowers (Supplementary Fig. 3a), (2) an increase in the 2PE emission ratio for the total volatiles in S-flowers (Supplementary Fig. 3b), and (3) no difference in the level of 2PE emission between W-flowers and S-flowers (Supplementary Fig. 3c). These results indicate that roses may synthesize 2PE *via* the novel pathway to compensate for the reduction in total volatiles caused by weak petal growth in the summer season. The total amount of 2PE per cell in S-flowers was several fold higher than that in W-flowers (Fig. 2b), but the total emission of 2PE per flower did not differ between S-flowers and W-flowers (see Supplementary Fig. 3c).

Plant pyruvate decarboxylases (PDCs) normally play a role in energy production through an ethanolic fermentative process under conditions of low oxygen. For example, *Arabidopsis thaliana PDC* (*AtPDC1*) and *AtPDC2* are induced by low oxygen stress^28^, and Mithran *et al.* showed that both AtPDC1 and AtPDC2 are involved in low oxygen tolerance^29^. Although microarray analysis indicates that there is a significant overlap in the expression profile between anoxic and heat responses, different responses of the *AtPDC* genes have been reported: *AtPDC1* is induced by both anoxia and a combination of anoxia and heat, whereas *AtPDC2* induction is observed in heat-treated seedlings, in addition to anoxia and a combination of anoxia plus heat^30^. Among the AtPDC1– AtPDC4 isoforms (Supplementary Fig. 4), AtPDC2 is the most similar enzyme to RyPPDC, and we confirmed the heat induction of *RyPPDC* in rose flowers (Fig. 2f). It seems that an as yet unidentified mechanism (*e.g. via* a transcription factor or small RNA) leads to RyPPDC and AtPDC2 induction in high-temperature conditions; furthermore, heat-responsive PDCs are likely have a different role from PDCs involved in anoxia tolerance. *AtPDC1* is dominantly expressed in seeds, whereas *AtPDC2* is detected in most organs^31^. Moreover, petunia PDC2 (PhPDC2) is characteristically expressed in pollen tubes and functions as a key enzyme in energy production through an alternative pathway comprising PDC, aldehyde dehydrogenase, and acetyl-CoA synthase^32^. We observed an accumulation of *RyPPDC* transcripts in the petal, as well as in the anther, calyx, rose hip, leaf, and stem (Supplementary Fig. 5). In addition to a role in volatile production, the up-regulation of RyPPDC in summer might contribute to energy complementation in weak growth organs where RyPPDC is expressed.

Several reports argue that PDCs are involved in the production of plant volatiles owing to changes in gene, protein and PDC activity^33^,^34^,^35^,^36^,^37^. High temperature induces AtPDC2^30^, banana PDC^35^ and *Citrus flavedo* PDC^38^, which shows the greatest similarity to RyPPDC (Supplementary Fig. 4). It is commonly known that plants have multiple PDC isoforms, such as AtPDC1–AtPDC4 in *A. thaliana*^31^, and PhPDC1 and PhPDC2 in *Petuni x hybrida*^32^. One of these isoforms may be PPA decarboxylase as seen in roses. Although our study was performed in rose flowers, the novel pathway (PPA-PAld-2PE) may also exist in other plants.

Our genetic analyses showed a lower level of *RyAAAT3* transcripts after 6 h but a continuous increase in *RyPPDC* transcripts in rose flower petals exposed to high temperature (Fig. 2f). This down-regulation of *RyAAAT3* expression may be explained by an accumulation of PPA in rose petals, as well as by negative feedback toward arogenate dehydratase in L-Phe biosynthesis^39^. In addition, the expression of transcription factors regulating floral scent production is altered at high temperature. High-temperature treatment of petunia flowers has been found to reduce the expression of genes relevant to the shikimate/phenylpropanoid pathway, including PAAS (corresponding to AADC in roses), and to up-regulate the expression of *EOBV*, a negative regulator of scent production, thereby resulting in a reduction of 2PE^40^. Our genetic analyses also indicated that the shikimate pathway (from shikimic acid to L-Phe), as well as the phenylpropanoid pathway (from L-Phe to 2PE), is affected in S-flowers. Next-generation sequencing (NGS) analysis also showed the increased expression of two transcription factor homologs in S-flowers (Supplementary Table 2): (1) RyODORANT1, which regulates the transcript levels of genes encoding shikimate pathway enzymes^41^; and (2) RyEOBI, a positive regulator of the phenylpropanoid pathway^42^. Conversely, S-flowers exhibited reduced expression of an EOBIII homolog (Supplementary Table 2), which is a negative regulator of floral scent production in the phenylpropanoid pathway^43^. Capillary electrophoresis (CE-MS) analysis revealed that the precursors of L-Phe, dehydroshikimic acid and chorismic acid, were decreased (Supplementary Fig. 6), indicating that the increased expression of *RyODORANT1* at high temperature up-regulates the shikimate pathway. Furthermore, the increased expression of *RyPPDC* was expected to be independent of the transcription factors EOBI and ODORANT1 because endogenous levels of PPA were decreased and 2PE levels were increased in S-flowers (Supplementary Fig. 6). The independent expression of *RyPPDC* was also suggested by the fact that changes in the transcript levels of the 2PE-relevant genes *AADC*, *RyAAAT3* and *PAR*, were not observed in S-flowers, although increased levels of *RyPPDC* were (Fig. 1f). Taken together, the genetic analyses suggest that two different genetic control mechanisms are potentially induced by high temperature to produce 2PE in S-flowers: (1) up-regulation of the shikimate pathway, leading to an increase in L-Phe (*via* the high expression of *RyODORANT1*); and (2) up-regulation of the availability of L-Phe (due to *RyODORANT1*) or the phenylpropanoid pathway (which does not involve the 2PE-relevant genes *AADC*, *RyAAAT3,* and *PAR*, and occurs *via* the increased expression of *EOBI* and decreased expression of *EOBIII*). The response of roses to high temperature is different to that of petunias in relation to 2PE production and the transcription factors ODORANT1 and EOBs.

The novel discovery of a summer alternative principal pathway for production of the scent compound 2PE leads to many interesting questions on the origin and evolution of alternative principal pathways. The origin of cultivated roses is unknown because roses have been cultivated in Europe and East Asia for several thousand years. Wild roses from Europe, Asia and North America have also been identified^44^. The alternative principal pathway may have originated in wild rose species as a heat adaptation when the species spread to low latitudes and/or low altitudes. Alternatively, this pathway may have been fixed during cultivation to produce blossoms in summer. Certain wild roses that grow in cool temperatures may not have this pathway. Questions also remain regarding the biosynthetic pathway of plant secondary metabolites. How is the alternative principal pathway established from biochemical reactions in relation to adaptations to heat shock? Why do roses exhibit an alternative principal pathway in summer? Is this additional pathway common for plant secondary metabolites? We should note that multiple biosynthetic pathways and bypasses are known for many essential metabolites because these metabolites are required for survival. However, these alternative pathways are usually not observed for seasonal adaptations but rather are compensatory pathways to manage lethal mutations and detrimental environmental damages. The findings presented here represent the first report of a seasonally activated alternative biosynthetic pathway that produces identical plant secondary metabolites.

In conclusion, this study provides biochemical and genetic evidence showing that roses produce the floral scent compound 2PE by two different biosynthetic pathways in response to seasonal changes in temperature. We also found evidence for the involvement of RyAAAT3 and RyPPDC in the alternative 2PE biosynthetic pathway. This discovery suggests that multiple pathways and the simultaneous activation of a seasonal alternative principal pathway toward a single end product are possible for important secondary chemicals in plants.

## Methods

### Plant materials and preparation of protoplasts

Cut flowers of *R. x hybrida* ‘Yves Piaget’ were purchased from Ichikawa Rosary in Mishima City, Shizuoka Prefecture, Japan. The stages of floral growth have been described previously^23^. In each experiment, we used GC-MS to analyze protoplasts that were isolated from W- or S-flowers and administered L-[^2^H_8_]Phe. Protoplasts were prepared as described previously^25^. In brief, the petals were sliced and immersed in enzyme-digestion buffer containing Cellulase and Macerozyme. The petal and enzyme mix was incubated for 3.5 h at 25 °C. After the petal tissues and cell debris were removed by nylon filter, the protoplasts were collected, washed and suspended in an appropriate volume of protoplast buffer.

### Chemicals and biochemicals

L-[2,3,3,2′,2′,4′,5′,6′-^2^H_8_]Phe (98 atom% ^2^H, Aldrich) was used in pulse-chase studies. The ratio L-[2,3,3,2′,2′,4′,5′,6′-^2^H_8_]Phe (L-[^2^H_8_]Phe)/L-[3,3,2′,2′,4′,5′,6′-^2^H_7_]Phe (L-[^2^H_7_]Phe)/L-[^2^H_6_]Phe was estimated to be 83/16.5/0.5 on the basis of ^1^H-NMR analysis, as reported previously^23^. Thus, a [^2^H_8_]-/[^2^H_7_]-2PE ratio of 83/17 should have been produced when no ^2^H atoms were abstracted or replaced with protons during the pulse-chase feeding experiments. The production ratios of [^2^H_8_]-/[^2^H_7_]-2PE were determined using the ion intensities at *m/z* 130 and *m/z* 129 for the molecular ions of [^2^H_8_]- and [^2^H_7_]-2PE, respectively. Abstraction of a ^2^H atom at the C3 (benzyl) position caused a decline in the ion intensity at *m/z* 98 [C_7_^2^H_7_]^+^. Accordingly, the ion intensities at *m/z* 98 and *m/z* 97 were used to confirm the chemical structures of [^2^H_8_]-2PE and [^2^H_7_]-2PE, respectively.

### Feeding experiments in protoplasts and intact flowers

L-[^2^H_8_]Phe and PPA were dissolved in protoplast buffer^25^ and added to the protoplasts at final concentrations of 2.5 μmol. The protoplasts were incubated at 30 °C for 24 h and volatiles were analyzed by GC-MS. In intact flowers, L-[^2^H_8_]Phe feeding of rose flowers has been described previously^23^.

### Assay of PPA decarboxylation activity

Reaction mixtures (200 μl) containing 5 mM PPA, 1 mM TPP, 5 mM MgCl_2_, and 100 μl of enzyme solution in a 0.1 M citrate buffer (pH 6.0) were incubated at 40 °C for 1 h. PAld was extracted with 300 μl of a mixture of hexane:ethyl acetate (1:1 v/v) from enzyme reacts and analyzed by GC-MS.

### Analysis of 2PE and PAld by GC-MS

Analyses of volatiles were performed by using a GC-MS QP5050 instrument (Shimadzu), controlled by a Class-5000 work station. For 2PE analysis, the GC was equipped with a capillary TC-WAX column (GL Sciences Inc., Japan) of 30 m × 0.25 mm I.D. and 0.25-μm film thickness. The column temperature was increased from 60 °C (3-min hold) to 180 °C (40 °C/min), and then to 240 °C (10 °C/min, 3-min hold). The injector temperature was 200 °C, the ionizing voltage was 70 eV, and the scanning speed was 0.5 scan/s, with an *m/z* range of 76–200. For PAld analysis, the GC was equipped with a capillary TC-5 column (GL Sciences Inc., Japan) of the same dimensions with a TC-WAX column; the column temperature was increased from 50 °C (3-min hold) to 90 °C (10 °C/min), then to 130 °C (30 °C/min), and finally to 290 °C (40 °C /min, 3-min hold). The injector temperature, ionizing voltage, scanning speed, and *m/z* range were the same as those for 2PE.

### Purification of RyPPDC

Flowers at stage 4 were harvested in August and ground in liquid nitrogen. The powder was extracted with extraction buffer with PVPP (Polyclar 10, ISP Japan). The buffer extracts were separated from debris and further purified by ammonium sulfate precipitation (20%–60%), HiTrap DEAE FF, HiTrap Phenyl HP, and Superdex 200 14/350. The detailed method is described in Supplementary Information. The peptide sequences of the purified protein were analyzed by Nano-LC-MS/MS (see Supplementary Information).

### Molecular cloning of *RyPPDC* and heterologous expression in HighFive cells

Total RNA was extracted from rose petals using the RNeasy Plant Mini Kit (Qiagen). cDNA of RyPPDC was amplified by RT-PCR using the 3′-RACE method with degenerate primers [TaKaRa RNA PCR Kit (AMV) Ver.3.0, TaKaRa]. 5′-RACE was performed with specific primers using the Smarter Race cDNA Amplification Kit (TaKaRa). The RyPPDC proteins expressed in insect cells were collected and washed in PBS^45^. The proteins were extracted with 100 mM Tris–HCl buffer (pH 8.0) containing 1% TritonX-100, 1 mM TPP, and 5 mM MgCl_2_. The recombinant proteins were subjected to further purification by HiTrap DEAE FF, HiTrap Phenyl HP, and Superdex 200 10/300 GL (GE Healthcare).

### Kinetic analysis for PPA and PA decarboxylation

The reaction components were as described above. For PPA decarboxylation analysis, the reaction was stopped by adding 300 μl of hexane:ethyl acetate (1:1 v/v) with 3-phenylpropionaldehyde (Wako Pure Chemicals) as an internal standard and anhydrous Na_2_SO_4_. For PA decarboxylation analysis, the reaction was stopped by adding 300 μl of 10 mM 2, 4-dinitrophenylhydrazine dissolved in methanol and derivatized at 40 ^ο^C for 1 h. After centrifugation, the supernatants were filtered (Millex LH, Millipore) and analyzed by HPLC. The separation module was equipped with a SHIMADZU SPD-20 A UV-VIS detector and a CAPSELL PAK C18-UG120 column (2.0 mm × 75 mm, SHISEIDO). The solvents were A (water) and B (acetonitrile). The column was developed by increasing the latter from 40% to 70% in 15 min, and then to 100% with a 5-min hold, at a flow rate of 0.2 ml/min at 40 °C. Detection wavelengths were 254 and 370 nm.

### Analyses of gene expression by qRT-PCR

Gene expression was measured by quantitative RT-PCR using a LightCycler 480 (Roche Applied Science) and specific primers. Primers are listed in Supplementary Table 3. The detailed analytical conditions are described in Supplementary Information.

### Temperature control for rose cut flowers

Rose petals were separated from rose flowers, and the bases were immersed in 10 mM L-[^2^H_8_]Phe. The petals were preincubated at 4 °C for 24 h and then further incubated at 4 °C or 30 °C during 24 h. 2PE was extracted by hexane for 24 h from the crushed petals in liquid N_2_.

### Next generation sequencer analysis

For RNA-Seq analysis of rose petals, we constructed cDNA libraries that were sequenced at the Kazusa DNA Research Institute, in Chiba, Japan. Total RNA extracted from W- and S-flower petals was individually subjected to mRNA purification and cDNA library construction using the TruSeq RNA Sample Prep Kit v2 (Illumina). The two cDNA libraries were sequenced by using Hiseq 1000 and Hiseq1500 sequencers (Illumina) with 100 bp paired-end (PE) reads. The Illumina reads were assembled by using CLC Genomics Workbench version 5.5 (CLC Bio) with the following parameters: minimum contig length, 350; perform scaffolding to obtain assembled contigs (FASTA file, data not shown). The resulting contigs were annotated by blasting against NCBI nr (non-redundant) protein databases and manually validated to obtain a finalized set of biosynthetic enzyme candidates involved in the shikimic acid pathway. The RPKM (Reads Per Kilobases per Million) values^46^ were calculated by using CLC Genomics Workbench version 5.5 (CLC Bio).

### Headspace sampling

Emitted volatiles were collected from rose flowers by dynamic headspace sampling and were sent through a glass tube filled with TENAX TA resin (60/80 mesh, 250 mg, Perkin Elmer) at 100 ml/min for 6 h^23^. The volatiles were analyzed by GC-MS connected to the Thermodesorption System (TurboMatrix ATD, Perkin Elmer). All peaks were assigned by reference to the NIST library.

### Analysis of endogenous metabolites by CE-MS and GC-MS

Cut roses at stage 5 (harvested in December and at the beginning of October) were incubated at 20 ^ο^C in a 12-h light/dark cycle under 6,000 lux. The petals were frozen in liquid nitrogen and stored at −20 ^ο^C. The crushed petals (50 mg) were homogenized using Multi-Beads Shocker (Yasuikikai, Osaka, Japan) at 2,000 × *g* for 10 s and extracted with 0.5 ml of ice-cold methanol containing 400 μM PIPES and methionine sulfone as an internal standard. Ice-cold Milli-Q water (0.5 ml) was added, and the sample was ultra-filtered through a 5-kDa cutoff filter (Millipore) at 9,000 × *g* for 10 min. The filtrate was analyzed by capillary electrophoresis-mass spectrometry (CE-MS) methods following previously published protocols^47^,^48^. All CE-MS experiments were performed by using the Agilent Capillary Electrophoresis System equipped with a 1100 series MSD mass spectrometer, a 1100 series isocratic HPLC pump, a G1603A CE-MS adapter kit, and a G1607A CE-ESI-MS sprayer kit (Agilent Technologies). CE-DAD experiments were performed by Agilent capillary electrophoresis with a built-in diode-array detector. The G2201AA Agilent ChemStation software for CE was used for system control, data acquisition and analysis, and MSD data evaluation.

Endogenous 2PE was determined by GC-MS. Lyophilized petals (0.5 g) were added to 10 ml of methanolic buffer [methanol:10 mM potassium phosphate buffer (pH 7.5) (8:2 v/v)] and homogenized on ice. Ethyl decanoate (0.21 μmol) and ribitol (5.46 μmol) were added as internal standards, and the volume was brought to 25 ml with Milli-Q water. For analysis of 2PE, the extracts were diluted 30-fold with Milli-Q water and applied to an HLB column (3 cc extraction cartridge, Waters). After washing and drying, the volatiles were eluted with 2 ml of hexane:ethyl acetate (1: 1 v/v) and analyzed by GC-MS.

### Statistical analysis

A two-tailed Student’s *t* test was used to test for statistical significance. *P* < 0.05 was considered statistically significant.

## Additional Information

**Accession codes:** RyPPDC (LC012788; R. x hybrida ‘Yves Piaget’).

**How to cite this article**: Hirata, H. *et al.* Seasonal induction of alternative principal pathway for rose flower scent. *Sci. Rep.* **6**, 20234; doi: 10.1038/srep20234 (2016).

## Supplementary Material

Supplementary Information
