# Supplementary material for: Seasonal induction of alternative principal pathway for rose flower scent
Source: Sci Rep. 2016 Feb 1;6:20234. doi: 10.1038/srep20234 (PMC4735289; doi:10.1038/srep20234)
Supplement: Supplementary Information [file srep20234-s1.pdf]

Title: Seasonal induction of alternative principal pathway for rose flower scent

Authors: Hiroshi Hirata[1†], Toshiyuki Ohnishi[2,3†], Kensuke Tomida[2], Haruka Ishida[2], Momoyo Kanda[2], Miwa Sakai[4], Jin Yoshimura[5,6,7,8], Hideyuki Suzuki[9], Takamasa Ishikawa[10], Hideo Dohra[3], Naoharu Watanabe[5,8*]

Affiliations:

[1] Graduate School of Science and Technology, Shizuoka University, 836 Ohya, Suruga-ku, Shizuoka 422-8529, Japan.

[2] Graduate School of Agriculture, Shizuoka University, 836 Ohya, Suruga-ku, Shizuoka 422-8529, Japan.

[3] Research Institute of Green Science and Technology, Shizuoka University, 836 Ohya, Suruga-ku, Shizuoka 422-8529, Japan.

[4] United Graduate School of Agricultural Science, Gifu University (Shizuoka University), 836 Ohya, Suruga-ku, Shizuoka 422-8529, Japan.

[5] Graduate School of Science and Technology, Shizuoka University, 3-5-1 Johoku, Naka-ku, Hamamatsu 432-8561, Japan.

[6] Department of Environmental and Forest Biology, State University of New York College of Environmental Science and Forestry, Syracuse, New York, 13210, USA.

[7] Marine Biosystems Research Center, Chiba University, Kamogawa, Chiba, 299-5502, Japan.

[8] Graduate School of Engineering, Shizuoka University, 3-5-1 Johoku, Naka-ku, Hamamatsu 432-8561, Japan.

[9] Kazusa DNA Research Institute, Kisarazu, Chiba 292-0818, Japan.

[10] Human Metabolome Technologies Inc., Tsuruoka, Yamagata 997-0052, Japan.

†These authors contributed equally to this article

*Corresponding author: Naoharu Watanabe

e-mail: watanabe.naoharu@shizuoka.ac.jp;

phone/fax: +81-54-238-4870

Graduate School of Engineering, Shizuoka University, Hamamatsu 432-8561, Japan.

Contact Information:

Hiroshi Hirata: xjtpt341@yahoo.co.jp; phone/fax: +81-54-238-4870

Toshiyuki Ohnishi: ohnishi.toshiyuki@shizuoka.ac.jp; phone/fax: +81-54-238-3082

Jin Yoshimura: jin@sys.eng.shizuoka.ac.jp; phone/fax: +81-54-238-4870

SUPPLEMENTARY INFORMATION

SUPPLEMENTARY METHODS

Purification of RyPPDC

Rose petals were ground in liquid nitrogen. The powder (approximately 100 g) was extracted with 1 l of buffer A [50 mM citrate buffer, pH 6.0, containing 1% polyoxyethylene (10) octylphenyl ether (Tryton X-100)] (Wako Pure Chemicals) in the presence of 100 g of PVPP (Polyclar 10, ISP Japan) and stirred for 2 h at 4°C. The buffer extracts were prepared from rose petals. After filtering with gauze, the filtrate was centrifuged (10,000 ×*g*, 30 min, 4°C). The supernatant (crude enzyme extract) was fractionated with ammonium sulfate. Proteins that precipitated in 20%–60% saturated ammonium sulfate were re-dissolved in approximately 40 ml of buffer B (20 mM potassium phosphate buffer, pH 7.5). This fraction was dialyzed in buffer B for 4 h at 4°C. The dialyzates were further purified by chromatography using a HiTrap DEAE FF column (5 ml, GE Healthcare) equilibrated with buffer C (20 mM potassium phosphate buffer, pH 7.5, containing 1 mM DTT and 1 mM TPP) and eluted with a linear 0–1.5 M NaCl gradient in the same buffer. Ammonium sulfate was added to the eluted fraction to a concentration of 20%, and the solution was applied to a HiTrap Phenyl HP column (5 ml, GE Healthcare) equilibrated with buffer D (50 mM potassium phosphate buffer, pH 7.0, containing 1 M ammonium sulfate, 1 mM DTT, and 1 mM TPP). The proteins were eluted in buffer D without ammonium sulfate in a stepwise manner: 20% buffer D for eight column volumes (CV), 35% buffer D for 5 CV, 50% buffer D for 10 CV, and 100% buffer D for 5 CV. Further purifications were performed using Superdex 200 14/350 column chromatography (CV 70 ml, GE Healthcare) equilibrated with buffer E (50 mM potassium phosphate buffer, pH 7.0, containing 0.15 M NaCl, 1 mM DTT, and 1 mM TPP). The resulting proteins were separated by SDS-PAGE (7.5% polyacrylamide gel) and visualized by Ag staining (Sil-Best Stain One, Nacalai Tesque, Kyoto, Japan). Peptide sequences were analyzed by Nano-LC-MS/MS.

Preparation and determination of peptide sequences by Nano-LC-MS/MS

Purified proteins were separated by SDS-PAGE, and the major bands were excised from the gel and destained with wash solution [25 mM NH_4HCO_3/acetonitrile (1:1 v/v)]. Proteins in the gel pieces were reduced and alkylated by respective treatment with 10 mM dithiothreitol/50 mM NH_4HCO_3 (45 min at 56°C) and 55 mM iodoacetoamide/50 mM NH_4HCO_3 (30 min at room temperature). After sequential washings with wash

solution and acetonitrile, the proteins were digested with trypsin (sequencing grade modified, Promega) at 37°C overnight. The tryptic peptides were extracted from the gel pieces with 50% acetonitrile containing 1% formic acid, and the extracts were pooled and concentrated in a vacuum centrifuge. The dissolved sample was centrifuged at 20,000 ×*g* for 10 min at room temperature, and the supernatant was subjected to LC-MS/MS analysis. Peptide assignments were performed using an LC-ESI-LIT-q-TOF mass spectrometer equipped with a Nano Frontier eLD System (Hitachi High-Technologies, Tokyo, Japan) and a nano-flow HPLC, NanoFrontier nLC (Hitachi High-Technologies). The LIT-TOF and CID modes were used for MS detection and peptide fragmentation, respectively. The trypsin-treated sample (10 μl) was injected, and the peptides were trapped on a C18 column, Monolith Trap (50 μm × 150 mm, Hitachi High Technologies). Peptide separation was achieved using a packed nano-capillary column (capillary-Ex nano mono cap, 0.05 × 150 mm, GL Science, Japan) at a flow rate of 200 nl/min. The separated peptides were then ionized with a capillary voltage of 1,700 V. The ionized peptides were detected at a detector potential TOF of 1,850 V. The peptides were eluted using an acetonitrile gradient (A: 2% acetonitrile containing 0.1% formic acid; B: 98% acetonitrile containing 0.1% formic acid; 0 min with A = 98%, B = 2%, followed by 60 min with A = 60%, B = 40%). All peptide mass data were analyzed by using Peaks software (Bioinformatics Solutions Inc.) and the MASCOT database (Matrix Science).

Analyses of gene expression by qRT-PCR

Total RNA was extracted from rose petals using the RNeasy Plant Mini Kit (Qiagen) according to the manufacturer's instructions. For qRT-PCR, cDNA was synthesized using the Quanti-Tect Reverse Transcription Kit (Qiagen). Averaged mRNA expression was normalized to β-actin expression. Serial dilutions of a standard solution were included for each gene to generate a standard curve and allow calculation of the input amount of cDNA for each gene. A LightCycler 480 system was incubated at 95°C for 10 min to activate the FastStart Taq DNA polymerase. The run conditions were 60 cycles at 95°C for 10 s, 55°C for 10 s, and 72°C for 10 s. Melting curves of each amplified gene were created to obtain PCR efficiency. Each gene was quantified on the basis of three independent reverse transcription reactions.

SUPPLEMENTARY TABLE AND FIGURE LEGENDS

Supplementary Table 1. LC-MSMS analysis of peptides derived from RyPDC

purified from rose petals. C[1] and M[2] mean carbamidomethylation of cysteine and oxidation of methionine, respectively.

Supplementary Table 2. Homologs involved in heat response and transcription factors for 2PE synthesis obtained by NGS analysis of rose flowers. S-flower/W-flower ratio was calculated by the average score of RPKM values.

Supplementary Table 3. Primers used in this study.

Supplementary Fig. 1. Purification and heterologous expression of RyPPDC. (**a**) Purification of RyPPDC protein from rose petals. (**b**) SDS-PAGE analysis of samples obtained from each purification step shown in (**a**). The proteins were extracted from rose petals harvested in S-flowers and separated on a 7.5% polyacrylamide gel. M, protein molecular marker purchased from Bio-Rad Ltd. The lanes were loaded with crude extracts (1), the ammonium sulfate precipitate (20%–60%) (2), a Hi-Trap DEAE FF fraction (3), a Hi-Trap Phenyl HP fraction (4), and the Superdex 200 p.g. fraction (5). (**c**) SDS-PAGE analysis of heterologously expressed RyPPDC for kinetics analysis. (**d**) Total ion traces from GC-MS analysis of PPA metabolites produced by recombinant RyPPDC extracted from insect cells.

Supplementary Fig. 2. Seasonal changes and relationship between [$^2H_7$]-2PE level and day length in *R. x hybrida* 'Yves piaget'. (**a**) Seasonal changes in the average day length in Mishima City. All of the data were obtained from the National Astronomical Observatory of Japan. (**b**) The relationship between the [$^2H_7$]-2PE ratio and average day length.

Supplementary Fig. 3. Seasonal changes in flower growth and floral scent emission in *R. x hybrida* 'Yves piaget'. (**a**) Seasonal change in the number of petals (closed circle), in total petal weight (opened circle) from a flower and in average temperature in Mishima city (red line). There was significant difference (P<0.05) in the number of petal between W-flowers (51.0 ± 1.9, n=5, means ± SE) and S-flowers (57.7 ± 1.4, n=5, means ± SE). In contrast, the total petal weight is significantly lower in S-flower (7.13 ± 0.56 g, means ± SE, n=5, from June to October) than in W-flowers (11.90 ± 0.88 g, means ± SE, n=5, from November to April) (P<0.01). That means petal size was lower in S-flowers (high temperature season) than W-flowers (low temperature season). (**b**) Proportions of emitted volatiles collected in W- and S-flower. Emitted volatiles were

collected by the dynamic head space sampling. (**c**) Amount of emitted 2PE in W- and S-flower. Statistically not significantly different between experiments. Error bars represent SE (n=4 in W-flower and n=15 in S-flower).

Supplementary Fig. 4. Phylogenetic tree of plant PDC and rose PPDC. The alignment was constructed using ClustalW 1.81. Abbreviations and accessions are as follows: ZmPDC (PDB: 1ZPD); *Zymomonas mobilis*, OsPDC1, 2 (AAA68290, A2XFI3); *Oryza sativa*, CsPDC (AAZ05069); *Citrus sinensis*, RyPDC (AB669188; *Rosa x hybrida* 'Yves Piaget'), RyPPDC (LC012788; *R. x hybrida* 'Yves Piaget'), PhPDC1, 2 (AY928612, AAX33299); *Petunia × hybrid*, PaPDC (ABZ79223); *Prunus armeniaca*, FaPDC (AAL37492); *Fragaria × ananassa*, AtPDC1-4 (AT4G33070, AT5G54960, AT5G01330, AT5G01320); *Arabidopsis thaliana*. ZmPDC was included in the alignment as an outgroup. Based on the alignment, a molecular phylogenetic tree was constructed by the neighbor-joining (NJ) method. The statistical significance of the NJ tree topology was evaluated by bootstrap analysis with 1,000 iterations. The tree was drawn with MEGA version 6[49,50].

Supplementary Fig. 5. Transcriptional analysis of *RyPPDC* in various organs of *R. x hybrida* 'Yves Piaget'. Total RNA was extracted from S-flower tissues. β-Actin was used as a housekeeping gene control. Expression levels in petal were set to 1.0. Error bars represent SE (n = 3).

Supplementary Fig. 6. Comparisons of endogenous metabolites in rose flowers harvested in W- and S-flower. (**a**) Schematic biosynthetic pathway to 2PE from dehydroshikimic acid. (**b**) Metabolites analysis in rose cut flower, W-flower (White box) and S-flower (Gray box). Error bars represent SE (n = 3). Symbols indicate $P < 0.05$ (*) and $P < 0.01$(**).

SUPPLEMENTARY REFERENCES

49. Tamura K, *et al.* MEGA5: molecular evolutionary genetics analysis using maximum likelihood, evolutionary distance, and maximum parsimony methods. *Mol Biol Evol.* **28**, 2731-2739 (2011).

50. Hall BG. Building phylogenetic trees from molecular data with MEGA. *Mol Biol Evol.* **30**, 1229-1235 (2013).

SUPPLEMENTARY TABLES

Supplementary Table 1

Amino acid sequence	Ion Score	Charge	Observed mass *m/z*
AILVQPDR	57.4	2+	456.2814
AVKPVMVGGPK	83.2	1+	1081.632
AVKPVM[2]VGGPK	78.0	1+	1097.627
DFLLGLAK	54.9	2+	438.7758
ELLEWGSR	40.7	2+	495.2708
IFVPDGHPLK	82.5	1+	1121.623
ILHHTIGLPDFSQELR	99.0	3+	626.0283
MGLEAAVEAAAEFLNK	97.2	1+	1662.829
M[2]GLEAAVEAAAEFLNK	93.9	2+	560.6279
NWNYTGLVDAIHNGEGK	75.4	3+	629.9933
VNVLFQHIQK	71.0	3+	409.2527
VRC[1]EEELIEAIETANGPK	63.4	3+	686.6997
VSAANSRPPNPQ	11.6	2+	619.3344
VTIGNGPAFGC[1]VLMK	96.9	2+	782.4313

Supplementary Table 2

Contig ID	Length	Description	Identity	E-value	S-flower /W-flower ratio	Significance
Contig00003111	783	EOBI [*Petunia x hybrida*] (AGC00813)	65%	6e-62	1.94	$P < 0.05$
Contig00008879	766	Emission of benzenoids III [*Petunia x hybrida*] (ADK12668)	64%	2e-106	0.64	$P < 0.05$
Contig00041094	1188	RecName: Full=Protein ODORANT1; AltName: Full=MYB-like protein ODO1 [*Petunia x hybrida*] (Q50EX6)	53%	3e-86	7.98	$P < 0.05$

Supplementary Table 3

Primers		Sequence (5′–3′)
3′-RACE		
	*RyPPDC*_3′-RACE	GCIATHCAYAAYGGIGARGG
5′-RACE		
	*RyPPDC*_5′-RACE	(5′-Phos)-GGTGGGCGGCTGTTGGCAGCAGAAACCC
Full length		
	*RyPPDC*_Forward	ATGGAACCTTCTACACTCA
	*RyPPDC*_Reverse	CTACTGAGGATTTGGAGGTC
Subcloning to pENTR/D-TOPO		
	*RyPPDC*_Forward	CACCATGGAACCTTCTACAC
	*RyPPDC*_Reverse	CTGAGGATTTGGAGGTCGGC
qRT-PCR		
	*AADC*_Forward	CCCCGAAGAGTTCAGGAAAC
	*AADC*_Reverse	GGTTCAACTCGGCTTAGAACTG
	*RyAAAT3*_Forward	TGGATGGCGGCTTGGTTGGT
	*RyAAAT3*_Reverse	TGAAGGTTGCAGGACCGCCC
	*RyPPDC*_Forward	AGCTCAGAAAGATGAACCTCTT
	*RyPPDC*_Reverse	ACAATTCTCAGGGAGGCGG
	*RyActb*_Forward	CTGAGGCTCCTCTTAACCCC
	Ry*Actb*_Reverse	CATACATAGCGGGCACATTG
	*PAR*_Forward	GACTTTGGCGGAGGATGCTG
	*PAR*_Reverse	AGCGTTGGCTGTAACAGAGG

Supplementary Figures

a

Step	Total protein (mg)	Specific activity (U/mg protein)	Total activity (U)	Purification fold	Recovery (%)
Crude extracts	739	0.91	670	1.0	100
Ammonium sulfate	624	0.22	137	0.2	84
DEAE sepharose	17.2	1.20	21.1	1.4	2.3
Phenyl sepharose	1.88	4.20	7.9	4.7	0.25
Superdex 200 p.g.	0.039	47	1.8	52	0.005

U: nmol/h

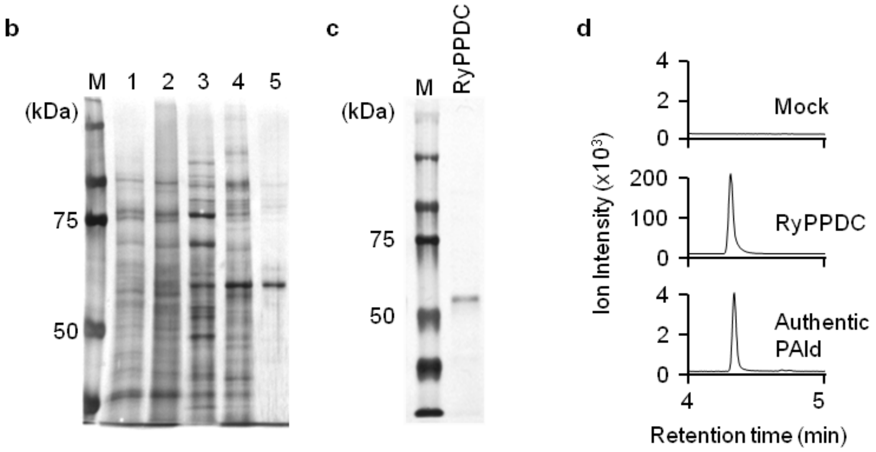

Supplementary Fig. 1

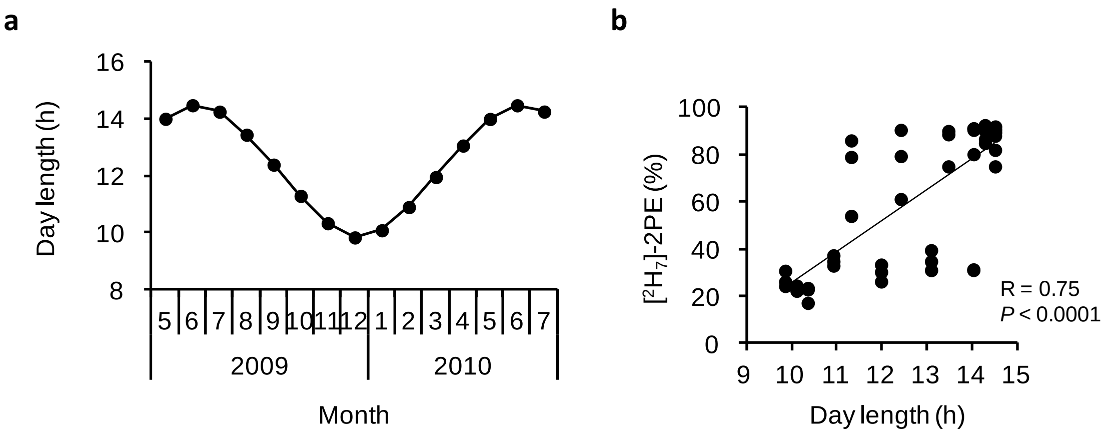

Supplementary Fig. 2

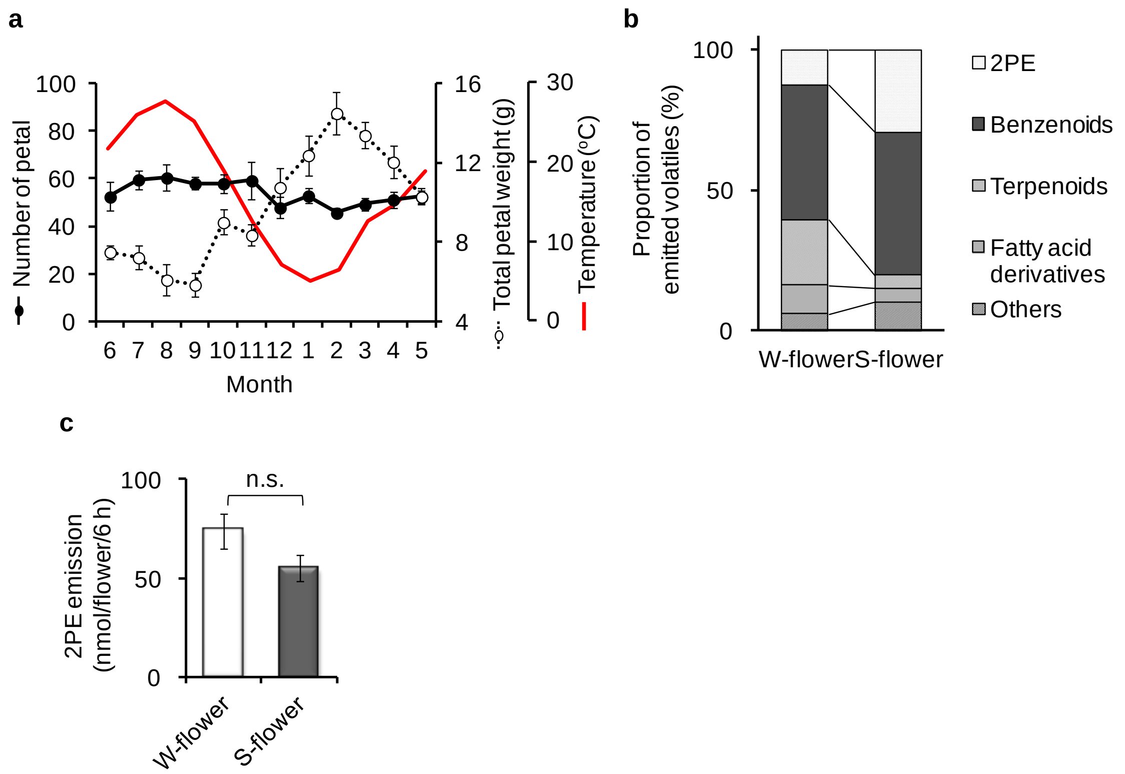

Supplementary Fig. 3

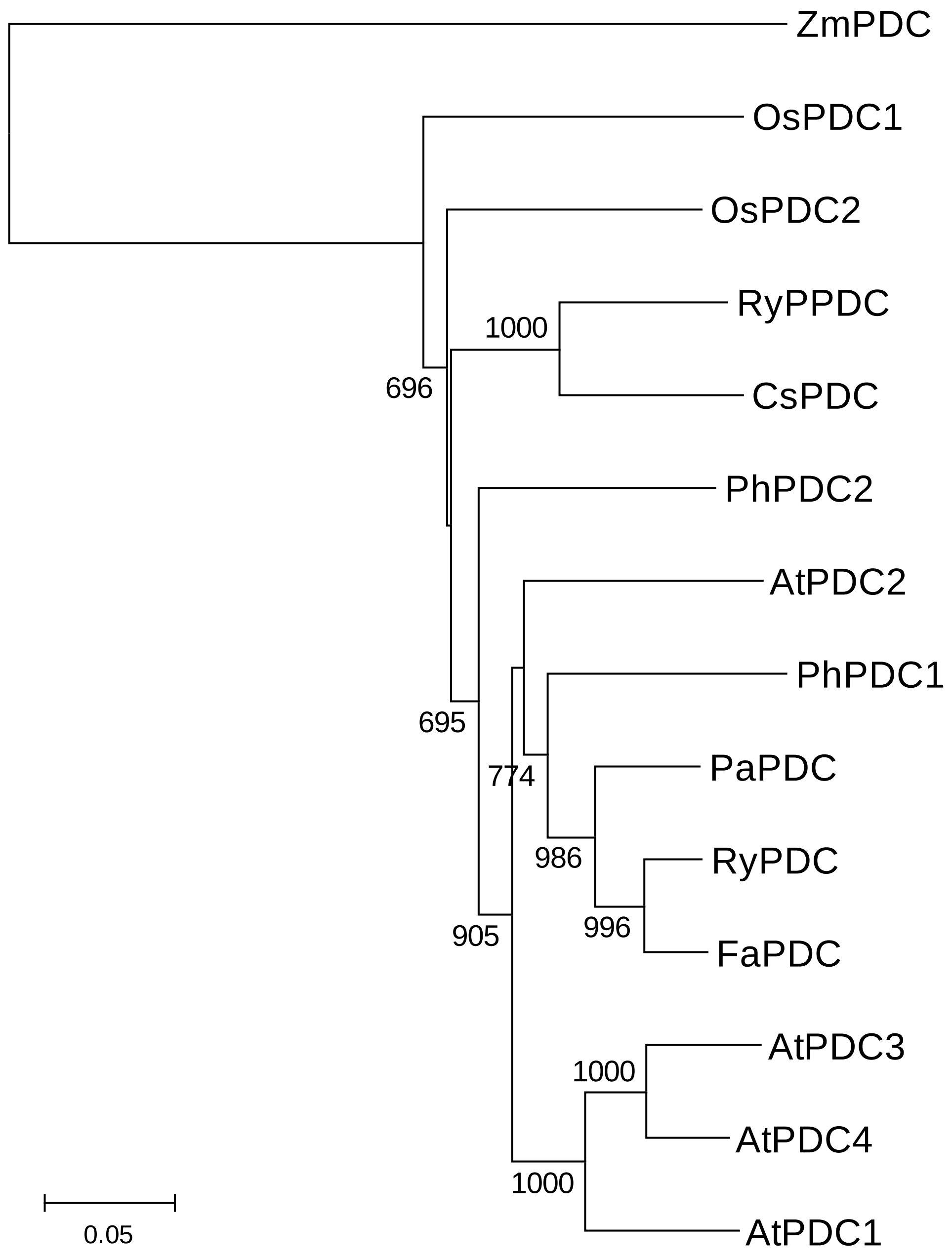

Supplementary Fig. 4

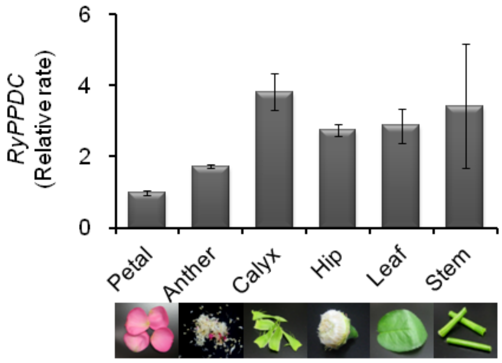

Supplementary Fig. 5

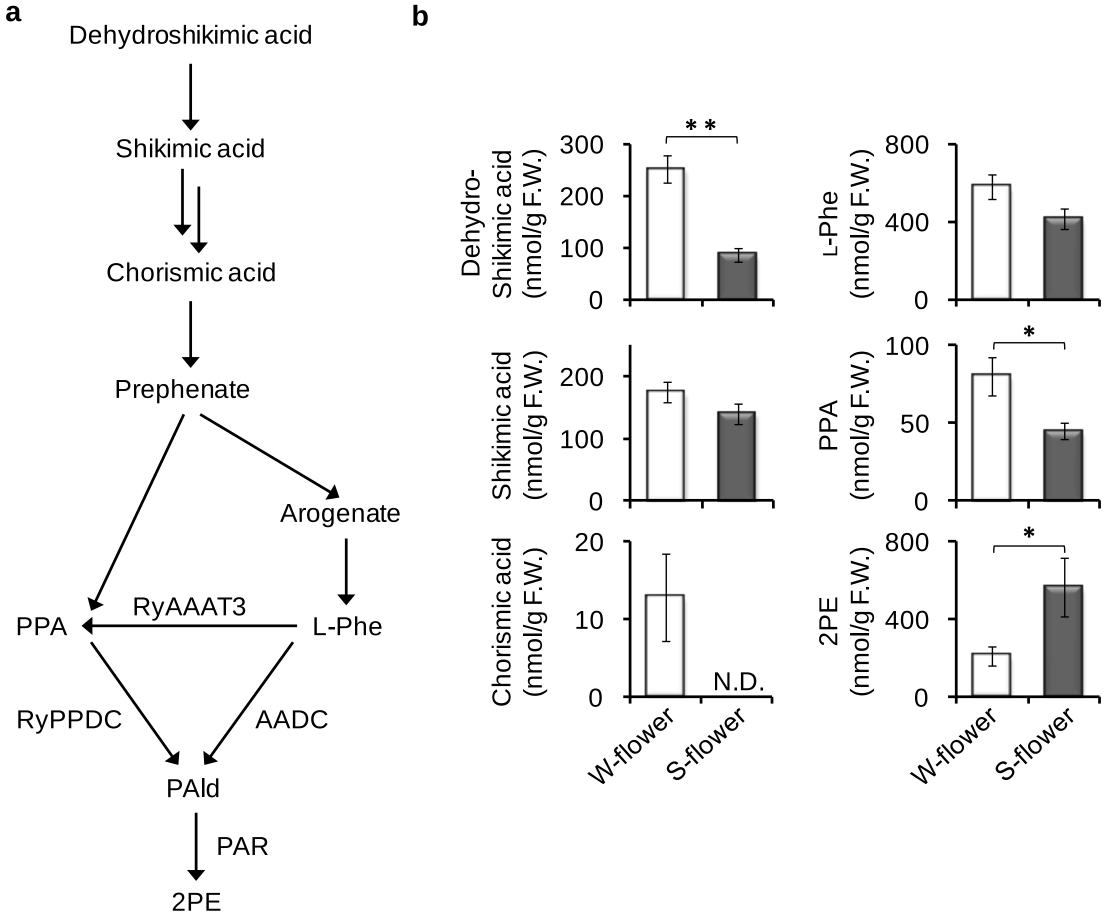

Supplementary Fig. 6
